# Living in a box: Understanding acoustic parameters in the NICU environment

**DOI:** 10.3389/fped.2023.1147226

**Published:** 2023-03-27

**Authors:** Christoph Reuter, Lisa Bartha-Doering, Isabella Czedik-Eysenberg, Marcus Maeder, Matthias A. Bertsch, Katharina Bibl, Philipp Deindl, Angelika Berger, Vito Giordano

**Affiliations:** ^1^ Musicological Department (Acoustics/Music Psychology), University of Vienna, Vienna, Austria; ^2^ Department of Pediatrics and Adolescent Medicine, Comprehensive Center for Pediatrics, Medical University of Vienna, Vienna, Austria; ^3^ Music Technology and Digital Musicology Lab, Institute for Musicology and Music Pedagogy, Osnabrück University, Osnabrück, Germany; ^4^ Department of Engineering Physics and Computation, School of Engineering and Design, Technical University Munich, Munich, Germany; ^5^ Department of Music Physiology, University of Music and Performing Arts Vienna, Vienna, Austria; ^6^ Division of Neonatology, Pediatric Intensive Care and Neuropediatrics, Department of Pediatrics and Adolescent Medicine, Comprehensive Center for Pediatrics, Medical University of Vienna, Vienna, Austria; ^7^ Department of Neonatology and Pediatric Intensive Care Medicine, University Children's Hospital, University Medical Center Hamburg-Eppendorf, Hamburg, Germany

**Keywords:** sound level, dB (A-weighted vs. unweighted), incubator, resonance, noise, NICU, preterm, acoustic features

## Abstract

**Background:**

In the last years, a significant body of scientific literature was dedicated to the noisy environment preterm-born infants experience during their admission to Neonatal Intensive Care Units (NICUs). Nonetheless, specific data on sound characteristics within and outside the incubator are missing. Therefore, this study aimed to shed light on noise level and sound characteristics within the incubator, considering the following domain: environmental noise, incubator handling, and respiratory support.

**Methods:**

The study was performed at the Pediatric Simulation Center at the Medical University of Vienna. Evaluation of noise levels inside and outside the incubator was performed using current signal analysis libraries and toolboxes, and differences between dB_A_ and dB_SPL_ values for the same acoustic noises were investigated. Noise level results were furthermore classed within previously reported sound levels derived from a literature survey. In addition, sound characteristics were evaluated by means of more than 70 temporal, spectral, and modulatory timbre features.

**Results:**

Our results show high noise levels related to various real-life situations within the NICU environment. Differences have been observed between A weighted (dB_A_) and unweighted (dB_SPL_) values for the same acoustic stimulus. Sonically, the incubator showed a dampening effect on sounds (less high frequency components, less brightness/sharpness, less roughness, and noisiness). However, a strong tonal booming component was noticeable, caused by the resonance inside the incubator cavity. Measurements and a numerical model identified a resonance of the incubator at 97 Hz and a reinforcement of the sound components in this range of up to 28 dB.

**Conclusion:**

Sound characteristics, the strong low-frequency incubator resonance, and levels in dB_SPL_ should be at the forefront of both the development and promotion of incubators when helping to preserve the hearing of premature infants.

## 1. Introduction

While on average, 0.1%–0.3% of all newborns suffer from hearing impairment or hearing loss, this rate is between 2% and 10% for preterm infants (1). By the age of 3 years, deficits in language acquisition are detectable in nearly 50% of very preterm infants (2, 3). It has been hypothesized that hearing impairment and subsequent language developmental problems may also be due to the increased noise levels preterm infants experience in their first weeks of life.

Noise levels measured in incubators are usually significantly higher than the 45 dB recommended by the American Academy of Pediatrics (4). In most cases, the baseline noise level in incubators is around 57 dB, and levels can increase to peaks of 91‒114 dB when the incubator is handled or opened (5). In the last years, a significant body of scientific literature was dedicated to the noisy environment preterm-born infants experience while they are fostered within neonatal intensive care units (NICUs) (6–9 etc.). In addition, basic research investigated noise levels and noise characteristics in NICUs (5, 10, 11), while clinical studies aimed at enhancing the situations for the infants in training staff (e.g., 12), using devices including ear muffs (e.g., 13), and improving incubators in terms of sound-damping (e.g., 10). Nevertheless, little is known about the character and interpretation of the noises: This concerns, first, the question of whether measurement in dB_SPL_ or dB_A_ is more appropriate, second, the question of computable sound characteristics beyond level measurement, and third, the question of the resonance characteristics of the incubator and its influence on the acoustic environment.

### 1.1. Sound level: “dB_A_ or not dB_A_, that is the question”

The sound pressure level in dB_SPL_ describes the sound level as a logarithmized ratio of measured sound pressure to a reference sound pressure (2 * 10^−5^ N/m^2^ respectively 2 * 10^−5^ Pa 14, p. 28). However, since human level perception is not only logarithmic but also frequency-dependent, the A-weighted sound level was introduced in 1936 to measure low-level noises (15). This weighting means that when sound levels are measured in dB_A_, the low frequencies (lower than 500 Hz) and high frequencies (higher than 5000 Hz) are given less weight, as it corresponds to the human auditory sensitivity and perception of quiet sounds. In this regard, levels measured in dB_A_ can be considered much lower than levels measured in dB_SPL_. This circumstance becomes essentially important, since the corresponding weighting curves have been identified in hearing tests with adults and the applicability for the acoustic environments in NICUs is hardly explainable (see below).

While some studies used dB_A_ units to investigate their research questions (e.g., 8–13, 16–21), only a few studies reported results in dB_SPL_ units (e.g., 6, 16, 20, 21). In some studies, both versions have been used (e.g., 5, 7, 24, 25). Even in the recommendations of the American Academy of Pediatrics (AAP) (4), there is no clear distinction between dB_A_ and dB_SPL_: Although in the mentioned official statement differences between dB_A_ and dB_SPL_ are briefly explained in the introduction, the manuscript provides mixed examples in dB_SPL_ and dB_A_ and makes a recommendation for a sound level below 45 dB, without clarifying if dB_A_ or dB_SPL_.

### 1.2. Timbre features: acoustic measurements beyond the dB scale

Previous studies have tried demonstrating the acoustical differences between an incubator and a womb. While noise in the incubator can rise sharply in the high-frequency range when using ventilatory support, noise in the womb predominantly consists of low frequency components (26). The incubator walls strongly attenuate sounds and voices from outside. In the womb, however, the sound transmission below 300 Hz almost corresponds to that in the air and above 300 Hz an attenuation of approx. 5 dB/Oct. sets in (27). Moreover, structure-borne excitation contacts with the incubator (like knocking, placing something on the incubator, opening or closing doors and shelfs) are strongly amplified in the incubator for a given resonance effect (28), while in the womb, mainly the mother's voice and other vibro-acoustic signals are transmitted through the mother′s body (27). In addition, the preterm neonate in the incubator is exposed to impulsive and abrupt sounds, contrary to the fetus in the womb. While up to now, almost only incubator levels have been measured, current signal analysis methods allow for comparatively measure many other acoustic properties in numerical values.

### 1.3. Resonance: there is something in the deep

Since the incubator is an air-filled cavity, resonances in the low-frequency range can be expected, boosting existing noise in the incubator. Contrarily, studies suggest that the cavity resonances in the womb are above 10 kHz and thus may not affect the hearing development of the fetus (29). Previous studies of sound levels in incubators have estimated strong resonance amplitudes in the frequency region of 125 Hz (20, 24, 25). If a more robust mathematical model confirmed this data, it would suggest once again a complete underestimation in an A-weighted level measurement (dB_A_) for all the reasons described above.

## 2. Aims

The aims of this study were:

 1. To compare real-life NICU noise measurements with already existing values reported in the literature.
 2. To describe sound characteristics in detail.
 3. To provide information about the resonance characteristics of the incubator cavity.

Specifically, we aimed to investigate differences between dB_A_ and dB_SPL_ values reporting dB_A_ and dB_SPL_ levels of several different noises typical for a NICU. In addition, we used standard signal analysis libraries to investigate the acoustic changes resulting from the incubator's structure, providing specific sound characteristics for each event considered. Moreover, since it can be assumed that the resonances of the incubator's cavity play a unique role regarding the acoustic environment of the premature infant, a simulation model was used to extrapolate specific information on the matter.

## 3. Methods

**Figure 1:**
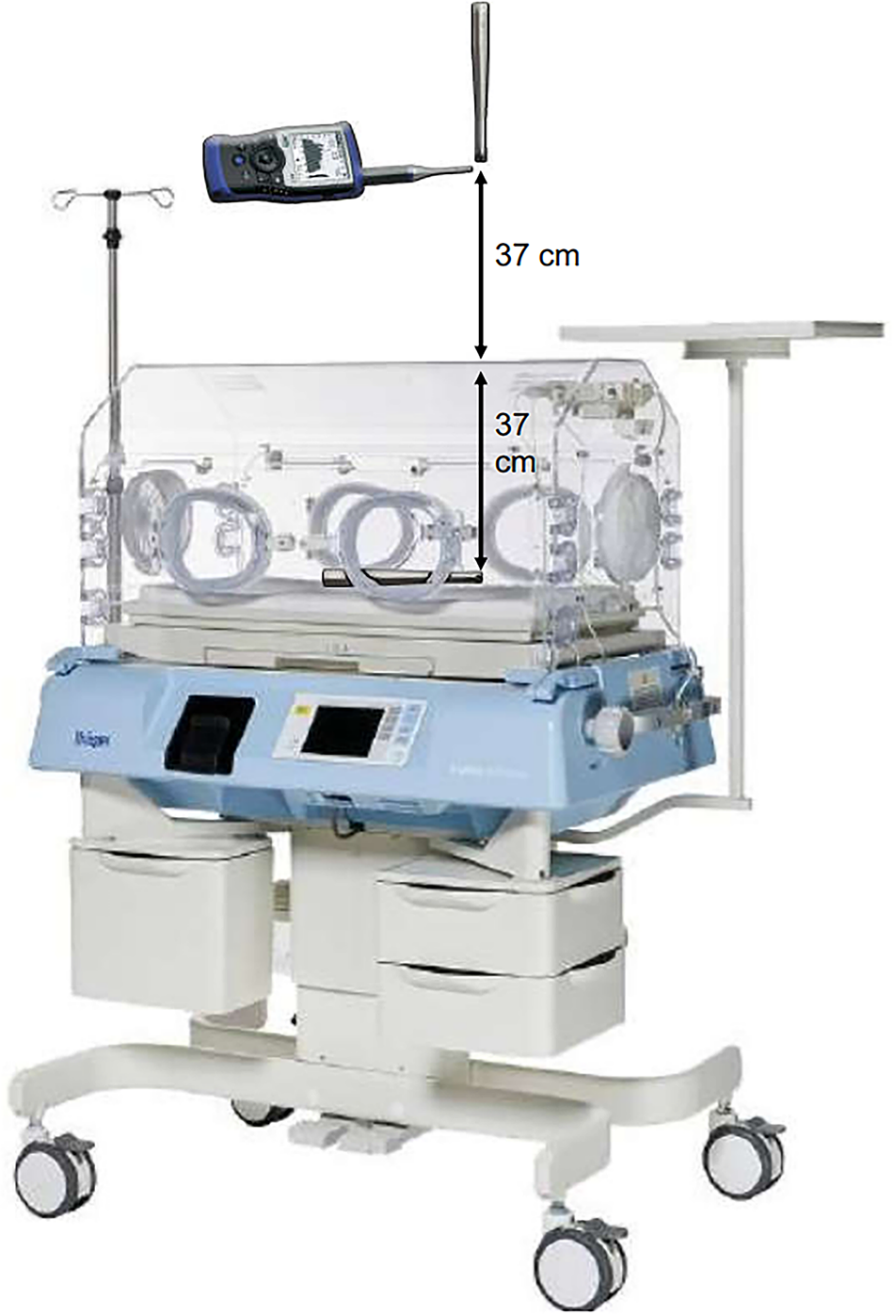
Microphone placement for level measurement inside and outside the Dräger Isolette 8000 incubator.

The study was performed at the Pediatric Simulation Center at the Medical University of Vienna. A simulation manikin (Paul, SIMCharacters® GmbH, Vienna, Austria) was placed within an incubator (Dräger Isolette 8000). An Esper K4 measurement microphone was set at the ear of the manikin inside the incubator (37 cm below the incubator ceiling) and outside the incubator (37 cm above the incubator; Figure 1). Both microphones were calibrated to 114 dB_SPL_ at 1000 Hz. In addition, level matching was performed with an NTi XL2 Acoustic Analyzer.

Sound level information was collected both in dB_SPL_ and dB_A_ (one single sound at a time) for the following main categories: 11 environmental noises (starting incubator engine, environmental noise (incubator OFF), environmental noise (incubator ON), normal conversation, light conversation, laughter, telephone, infusion pump alarm, monitor alarm (anomaly), monitor alarm (emergency), blood pressure, 12 incubator handlings (water flap, pouring water into the incubator, incubator doors opening properly, incubators doors closing properly, incubator doors closing improperly, hatch closing, hatch opening, incubators drawer, neighbor incubator doors closing (1.82 m distance), taking a stethoscope from incubators wall, putting a stethoscope on the incubator, suctioning tube, and six levels of respiratory support (CPAP Nasal Prongs, red, 2, 4, 6, 8, 10, 12 L/min). Statistical differences between measurements inside and outside the incubator, and between dB_SPL_ and dB_A_, respectively, were calculated using paired t-tests. Data were further compared to existing values reported in the literature.

Timbre features for sounds generated inside and outside the incubator were evaluated using current signal analysis libraries and toolboxes. The MIRtoolbox (30) and the Miningsuite in Matlab (31) as well as Essentia (32), Librosa (33), the AudioCommons Timbral Models (34) in Python as well as HEAD acoustics ArtemiS Suite were used to collect more than 70 temporal, spectral and modulatory timbre features as numerical values for each recorded sound for timbre comparisons between sounds recorded inside vs. outside the incubator. Statistical analysis (*t*-tests for sound feature values measured inside vs. outside with *p* < 0.05) was performed with JASP and data visualization *via* Plotly.js.

Moreover, in order to obtain resonance information, the impulse response of the incubator cavity was spectrally analyzed, and the result was compared with the impulse response calculated from the room volume of the incubator cavity using the finite element method in the commercial simulation software Abaqus. This software was also used for simulations and post-processing when calculating the full coupling between the cavity pressure and structural vibrations.

## 4. Results

### 4.1. Comparison of noise measurements to previously reported sound levels

Noise level results of the present study were ranked within previous studies’ results. Table 1 displays the results of environmental, handling, and respiratory noise levels for dB_A_ and dB_SPL_ separately (all audio files as well as a *t*-test on the level differences inside and outside the incubator are available at https://muwidb.univie.ac.at/incubator/).

**Table 1:** Measured noise values in dB_SPL_ and dB_A_ within the simulation room and incubator.

Measurement	dB_SPL_ (inside incubator)	dB_A_ (inside incubator)	dB_SPL_ (outside incubator)	dB_A_ (outside incubator)
***Environment***				
Starting incubator engine	82.05	73.64	76.18	75.34
Environmental noise (incubator OFF)	67.56	59.42	63.39	50.86
Environmental noise (incubator ON)	67.56	59.42	63.39	50.86
Normal conversation	78.04	73.4	84.39	84.04
Light conversation	73.77	58.44	71.07	66.16
Laughter	80.98	75.65	86.65	89.21
Telephone	67.68	59.46	84.65	77.6
Infusion pump alarm	67.09	58.59	70.2	66.72
Monitor alarm (anomaly)	68.16	59.46	67.98	63.22
Monitor alarm (emergency)	69.65	60.02	67.83	62.34
Blood pressure measurement	66.02	58.12	68.04	67.27
***Handling***				
Water flap	87.69	82.68	84.38	82.04
Pouring water into incubator	74.15	66.67	72.1	72.73
Incubator doors opening properly	73.25	71.43	63.5	60.39
Incubators doors closing properly	80.85	74.99	63.81	58.46
Incubator doors closing improperly	100.98	100.31	86.92	86.73
Hatch closing	92.52	87.77	85.72	85.56
Hatch opening	79.34	74.78	67.14	62.5
Incubators drawer	84.31	83.93	83.79	80.08
Neighbor incubator doors closing (1.82 m distance)	79.12	69.78	81.88	80.34
Taking stethoscope from incubators wall	87.43	82.99	68.78	60.31
Putting stethoscope on incubator	94.99	94.86	88.95	86.47
Suctioning tube	93.04	91.33	76.05	73.55
***Respiratory support***				
Respirator 12l min	93.13	84.69	66.74	58.3
Respirator 10l min	89.96	77.73	66.49	54.26
Respirator 8l min	84.72	83.51	64.62	59.71
Respirator 6l min	80.58	80.72	64.89	61.24
Respirator 4l min	62.83	63.17	62.83	55.29
Respirator 2l min	64.14	63.83	64.14	54.05

**Figure 2:**
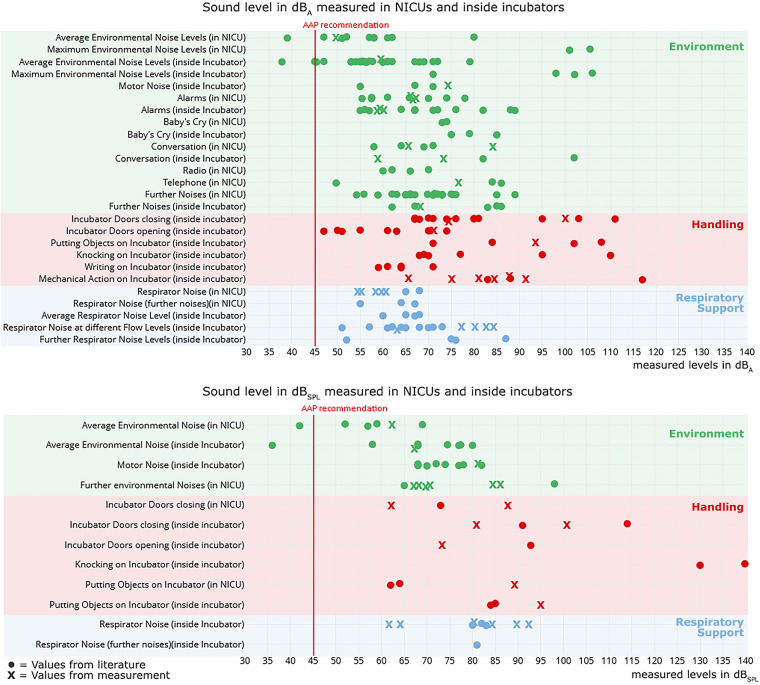
Measured noise level values in dB_A_ (top) and dB_SPL_ (bottom) compared to noise level values from literature related to the environment (green), handling (red) and respiratory support (blue) (noise level values have been taken from 6–13, 16–25, 33).

Both previous and present studies’ measurements revealed mostly values above 45 dB (Figure 2). In sum, more studies reported values in dB_A_ than dB_SPL_ levels. Nevertheless, similarly to our investigation, dB_SPL_ values were considerably higher than the corresponding dB_A_ values (see Table 2).

**Table 2:** Mean values and standard deviations as well as the difference of the mean values between the measured dB_SPL_ and dB_A_ values.

Measurement inside incubator	Environment	Handling	Respiratory support
dB_SPL_ (mean)	71.69	85.64	79.23
dB_SPL_ (Standard deviation)	5.71	8.29	11.81
dB_A_ (mean)	63.24	81.79	75.61
dB_A_ (Standard deviation)	6.77	10.1	8.84
difference (mean, dB_SPL_-dB_A_)	8.45	3.85	3.62
Measurement outside incubator	Environment	Handling	Respiratory support
dB_SPL_ (mean)	73.07	76.92	64.95
dB_SPL_ (Standard deviation)	8.17	9.07	1.34
dB_A_ (mean)	68.51	74.1	57.14
dB_A_ (Standard deviation)	11.67	10.57	2.77
difference (mean, dB_SPL_-dB_A_)	4.56	2.82	7.81

All situations/actions considered far exceed in most of the cases the recommended level proposed by the AAP (4).

### 4.2. Sound levels occurring inside and outside the incubator

The levels of environmental noises inside the incubator were not significantly different from those recorded outside the incubator, both for measurements in dB_A_ and dB_SPL_ (Table 3). However, handling in and around the incubator, resulted in significantly higher levels of noise inside than outside the incubator, with noises exceeding 100 dB_A_ and dB_SPL_, respectively. Finally, respiratory support yielded significantly higher noise levels within the incubator than outside. Importantly, all measurements showed a significant difference in noise levels measured in dB_A_ vs. measurements in dB_SPL_.

**Table 3:** *t*-test comparing ranked NICUs noises.

Measurements	Inside the incubator	Outside the incubator	Statistics t (*p*)
**Environment**			
dB_SPL_ mean (SD) (range)	71.69 (5.99)	73.07 (8.67)	0.70 (0.502)
	66.02–85.05	63.39–86.65	
dB_A_ mean (SD) (range)	63.24 (7.10)	68.51 (12.24)	2.09 (0.063)
	58.12–75.65	50.86–89.21	
Statistics t (*p*)	**10.33 (0.000)**	**3.14 (0.011)**	
**Handling**			
dB_SPL_ mean; SD (range)	85.64 (8.66)	76.92 (9.47)	**4.23 (0.001)**
	73.25–100.98	63.50–88.95	
dB_A_ mean; SD (range)	81.79 (10.54)	74.10 (11.04)	**2.68 (0.021)**
	66.67–100.31	58.46–86.73	
Statistics t (*p*)	**4.23 (0.001)**	**2.68 (0.021)**	
**Respiratory support**			
dB_SPL_ mean; SD (range)	79.23 (12.94)	64.95 (1.47)	**3.01 (0.030)**
	62.83–93.13	62.83–66.74	
dB_A_ mean; SD (range)	75.61 (9.68)	57.14 (3.03)	**5.79 (0.002)**
	63.17–84.69	54.05–61.24	
Statistics t (*p*)	**1.65 (0.159)**	**6.00 (0.002)**	

Bold values indicate the t-value of the t-test (the t-value is the size of the difference relative to the variation in the sample data).

### 4.3. Evaluation of the timbre features

**Figure 3:**
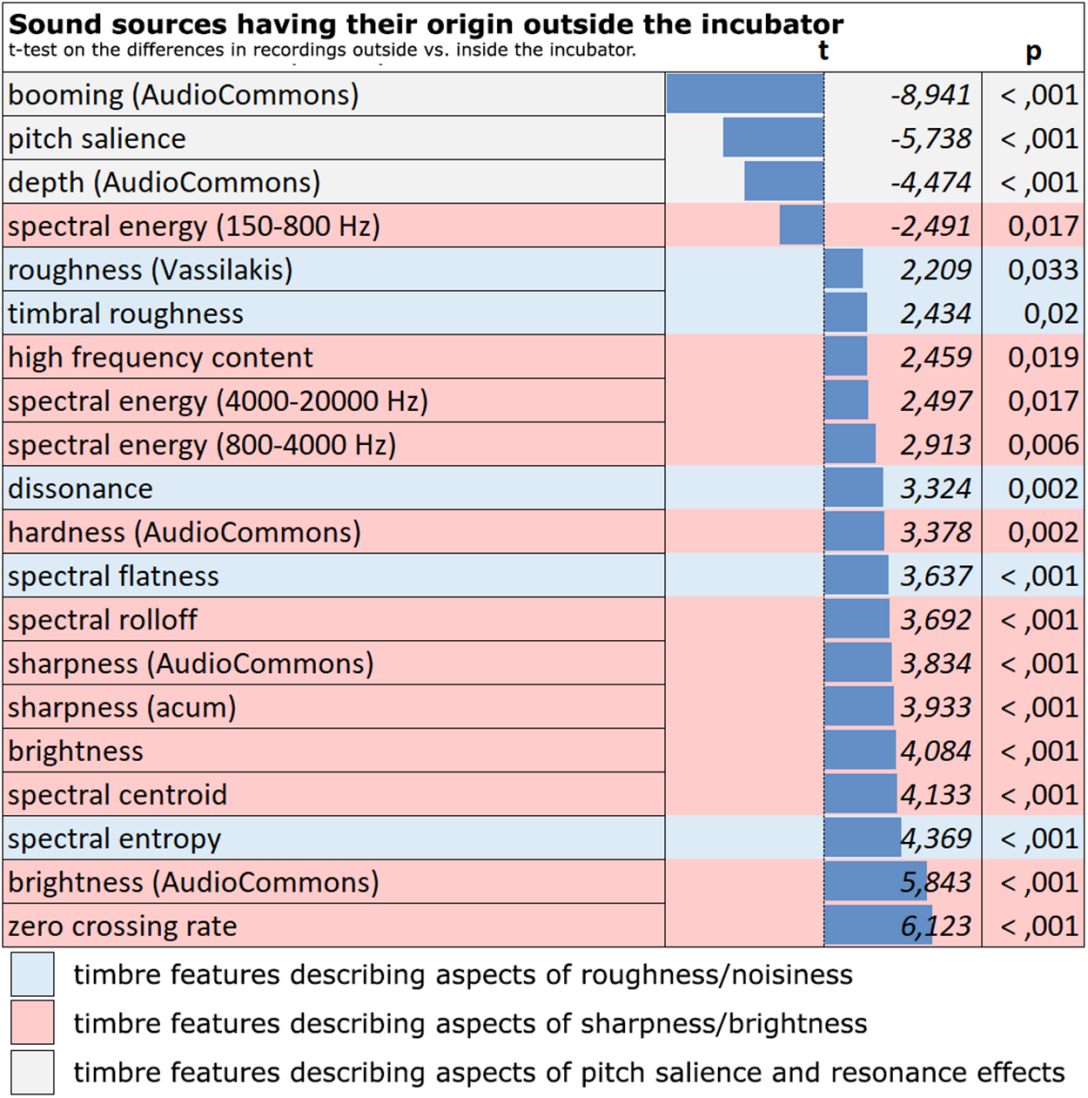
Noises from outside the incubator. Timbral differences between the recordings outside and inside of the incubator (marked red: timbre features describing aspects of sharpness/brightness; marked blue: timbre features describing aspects of roughness/noisiness, marked grey: timbre features describing aspects of pitch salience and resonance effects).

Most of external sound sources lead to significant differences in timbre features outside vs. inside the incubator (Figure 3). Evaluation of the timbre features of the collected sounds revealed that the effects of the incubator box on noises coming from sound sources outside the incubator could be described quite well using three characteristics bundles of timbre features describing the sharpness/brightness, the roughness/noisiness and the pitch salience/resonance effects (see Figure 3):

External noises sound duller (less bright/sharp) and less harsh/rough or noisy inside the incubator. Furthermore, they take on a more tonal character inside the incubator, which—like a stronger booming—is an indication of the incubator box's sound-shaping resonance (see below).

**Figure 4:**
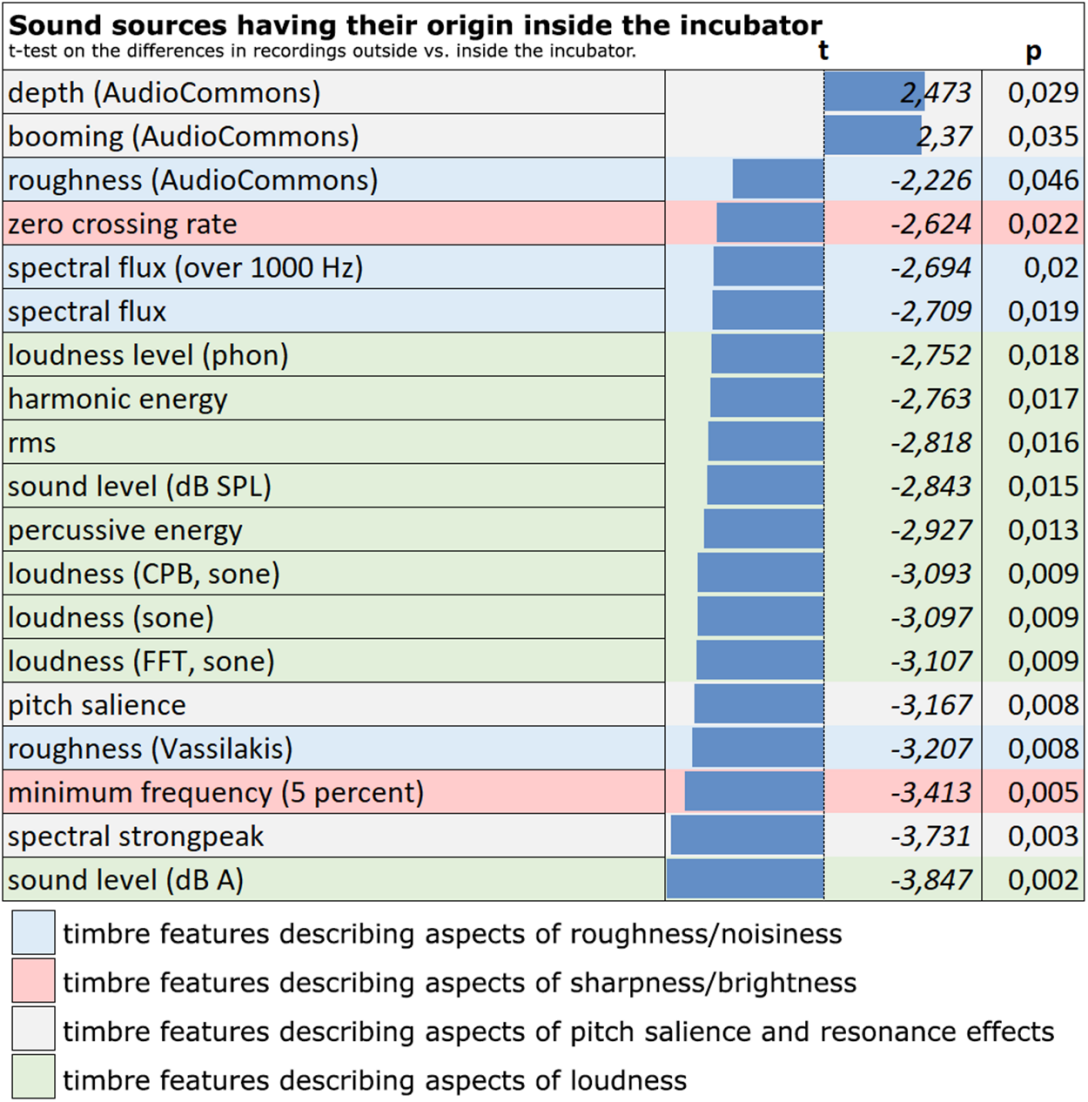
Noises from inside the incubator. Timbral differences between the recordings inside and outside of the incubator (marked red: timbre features describing aspects of sharpness/brightness; marked blue: timbre features describing aspects of roughness/noisiness, marked grey: timbre features describing aspects of pitch salience and resonance effects, marked green: timbre features describing aspects of loudness).

For sounds that originate inside the incubator, such as ventilation or suction sounds, the most significant differences between inside and outside the incubator are—besides strong loudness differences—again evident in the timbre features describing sharpness/brightness, roughness/noisiness and pitch salience/resonance effects (Figure 4).

Sounds that originate inside the incubator sound duller and less rough/noisy on the outside than on the inside, but are more tonally shaped by the resonance of the incubator box. The significant difference in loudness between inside and outside is also particularly apparent. I.e., the volume occurring inside (especially due to the ventilation support) is not perceived as loud at all by a listener from the outside (see below).

### 4.4. Resonance

**Figure 5:**
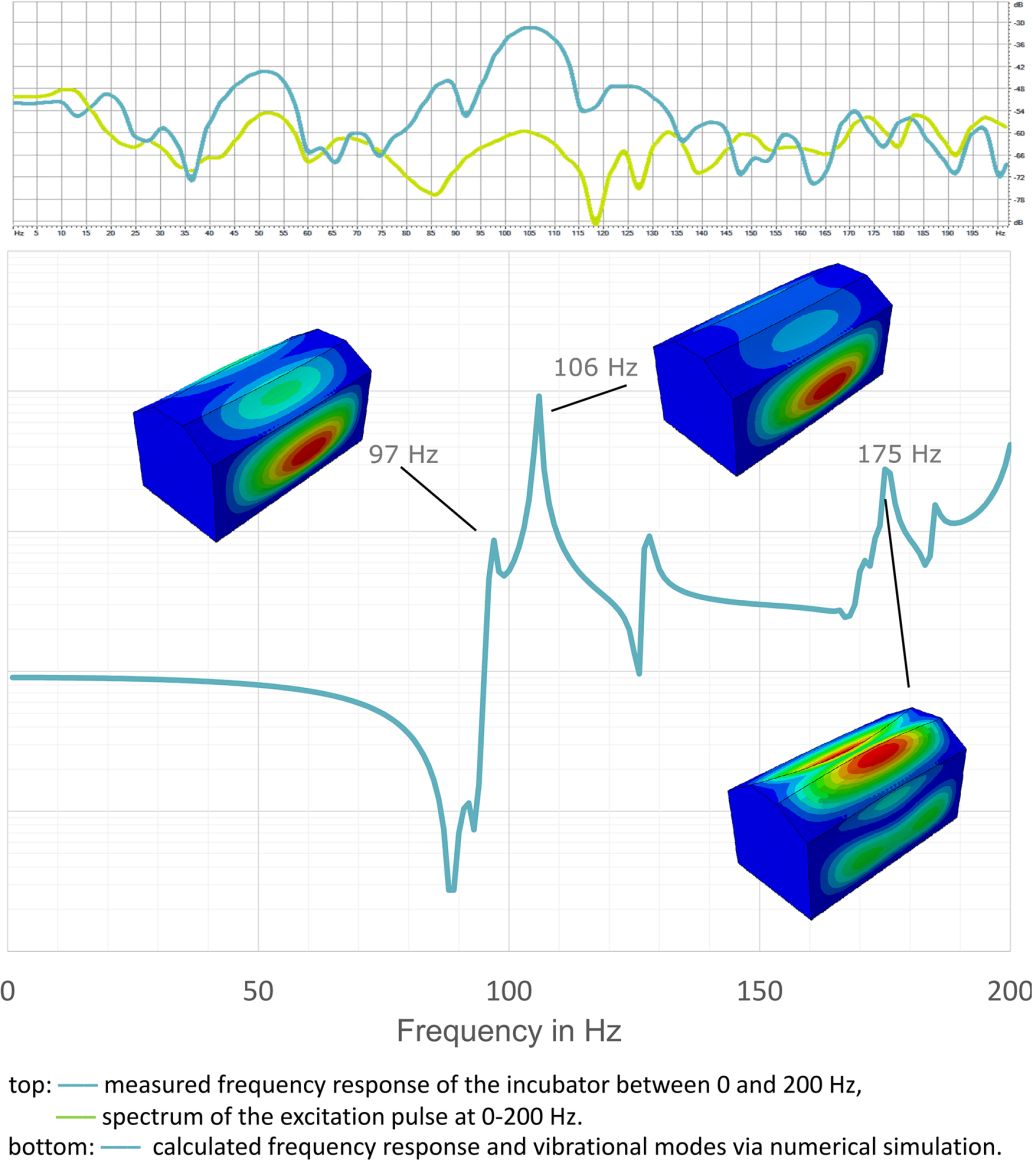
Measured (top) and simulated (bottom) resonance characteristics of the incubator.

Within the incubator, a main resonance could be measured at 97 Hz. Here, an amplification of the sound by 28 dB takes place. This main resonance was found both in the actual measurement and in the numerical simulation of the incubator box (34). In addition, two further resonances at 106 and 175 Hz were found in the simulation. The acoustic behavior of an incubator was successfully modeled based on acoustic and geometric measurements using the Finite Element Method. The simulations show coupled acoustic and vibrational modes in the low-frequency regime following the acoustic measurements (Figure 5).

## 5. Discussion

Our results confirm previous studies revealing high noise levels within the NICU environment. We confirm the high noise level related to real-life situation inside and outside the incubator. Most of the noise situations described in this manuscript far exceed not only the recommendation of the AAP but also international guidelines provided by the WHO [recommending noise level not exceeding 35 A-weighted decibels (dB_A_) during the day and 30 dB_A_ at night] and the United States Environmental Protection Agency [recommending daytime and nighttime sound levels of less than 45 and 35 dB (A), respectively] (35, p. XIV and 44, 36). These levels should be respected as much as possible because they could impact patients’ well-being, particularly in an intensive care setting (36). The present study exhibits significant differences between measurements in dB_A_ and dB_SPL_ values. Pitch salience, booming, spectral centroid, and spectral bandwidth were identified as the most important features to describe sound characteristics inside and outside the incubator. A mathematical model identified resonances with maximal levels at 97 and 127 Hz.

### 5.1. dB_A_ or dB_SPL_ for noise level measurements in incubators?

Sound measurements with A-weighting consider the frequency-dependent sensitivity of the ear along the 40-phon curve (15). However, the A-weighting does not fully correspond to the 40-phon curve. Broadband noise measured with A-weighting gets underestimated by 10–20 dB compared to a phon-measurement (37). Comparisons of the 40-phon curve and the A-weighting curve show that both low-frequency levels (below 400 Hz) and high-frequency levels (above 5000 Hz) are lower when measured in A-weighting than they are in both the 40-phon curve and the dB_SPL_ level. Thus, noise levels measured in dB_A_ are misleading as A-weighting is designed to measure low levels. Measurements of medium (>50 dB) or strong noise levels (>80 dB), as they occur in incubators, in A-weighting are incorrect, as at stronger levels the proportions of high and low frequencies are essentially underestimated (39). Significant differences between dB_A_ and dB_SPL_ were detected in the records performed for this study as well as when comparing our results with already existing literature. These observations should bring awareness to performing future studies and interpreting already existing ones on this topic. Moreover, it must be clarified that the hearing threshold, and along with it, the phone curves (or equal-loudness contours) of newborns are by no means the same as that of an adult human: While the outer ear canal resonance in adult humans is about 2‒4 kHz, it is about 6 kHz in newborns (38), resulting in a shift of the equal-loudness contours in newborns. To that end, also from this perspective an A-weighting of noise level measurement for incubator sounds can be considered meaningless or having little informative value, since up to now, no reliable investigations on the hearing sensitivity and perception of newborns have been reported.

Nevertheless, with this knowledge, two points need to be considered that have been more or less ignored so far in previous research:

1) For adults, exposure to 85 dB over 8 h per day is considered to cause a permanent hearing threshold shift (e.g., 14, p. 499). While the hearing threshold of adults is particularly sensitive between 2 and 4 kHz and the most sensitive point in the hearing threshold of newborns can be assumed at about 6 kHz (38), sound sources in the incubator environment whose spectral maxima are in the 6 kHz range are particularly dangerous here. These are mainly found in the respiratory support. According to our measurements, the respiratory support starts to be risky for the hearing health at a flow rate of 8 L/min at 84.72 dBSPL with an increase to 89.96 dB at 10 L/min and 93.13 dB at 12 L/min.
2) If the natural resonance of the outer ear canal is about 6000 Hz according to Kruger (38), then it must be remembered that this value applies to the transmission of airborne sound. In its natural environment, the embryo is surrounded by water, which is why, for the calculation of the intrinsic resonance of the outer ear canal, we can no longer assume a sound velocity of approx. 343 m/s at 20°C, but a sound velocity in water of approx. 1.484 m/s at 20°C. This means that at a resonance of 6000 Hz, the wavelength in the air is 5.7 cm. For the tube of the outer ear canal, which is closed on one side by the ear drum, this means that it must be 5.7/4 = 1.425 cm long in order to have 6000 Hz as its resonance frequency. If we now calculate the natural resonance of this tube with a wavelength of 5.7 cm with a speed of sound in water of approx. 1.484 m/s, we obtain 26035 Hz. This resonance frequency is far above human hearing capabilites. In other words: In the amniotic fluid in the womb, the resonance frequency of the outer ear canal does not contribute to an excessive sensitivity of the hearing threshold, but in the air it does. And it is precisely there that the high-frequency component of the respiration supply hits a particularly sensitive area of the hearing threshold mostly for longer time during the stay in the incubator.

### 5.2. Resonance and main sound characteristics within the incubator

The low-frequency main resonance (here at 97 Hz), which strongly effects incubator boxes, can hardly be captured in measurements using an A-weighting. The influence of the damping and inherent resonances of the incubator box is particularly evident in the change of sound characteristics when comparing sounds measured inside and outside the incubator: Noises from the outside sound more tonal inside the incubator, booming and muffled as well as less rough or noisy (increased pitch salience and booming, as well as lowered sharpness/brightness inside the incubator). Similar holds for sounds that occur inside the incubator. To a listener outside the incubator, they appear quieter but also more damped and less noisy. The resonance at about 125 Hz already observed by Seleny (20), Falk and Farmer (24) and Blennow et al. (25) could be found in the present study at 97 Hz. It can be assumed that the resonance observed in the former studies was also in this range, but that it was located at 125 Hz, since, in these studies, the sound analysis was performed with octave bandpass filters that were common at that time and whose center frequency in this frequency range was at 125 Hz. The fact that this booming resonance, which strongly influences the sound, was undetectable in most previous measurements is also due to the A-weighting of sound level measurement, where the low frequencies are attenuated in their levels: While sound levels have been measured in dB_A_ in the most incubator noise studies, Seleny (20), Falk and Farmer (24), and Blennow et al. (25) were among the few authors to measure sound level in dB_SPL_.

## 6. Conclusion

The measured values not only provide insight into the level differences inside and outside the incubator (50‒100 dB_A_ resp. 62‒101 dB_SPL_) and reveal the timbral differences caused mainly by the damping and the self-resonance of the incubator box.

Timbre features describing aspects of sharpness/brightness, roughness/noisiness, pitch salience, and loudness as well as the strong (up to 28 dB) low-frequency incubator resonance, and levels in dB_SPL_ (instead of dB_A_) should be at the forefront of both the development and promotion of incubators when helping to preserve the hearing of premature infants. To that end, we would like also to underline the importance of an adequate exposure to sound in the extra uterine environment, rather than a complete deprivation. Increasing NICU staff-awareness could contribute to this goal as also the development of new technologies.

## Data Availability

The original contributions presented in the study are included in the article/**Supplementary Material**, further inquiries can be directed to the corresponding author.
