# Supplementary material for: Living in a box: Understanding acoustic parameters in the NICU environment
Source: Front Pediatr. 2023 Mar 27;11:1147226. doi: 10.3389/fped.2023.1147226 (PMC10083238; doi:10.3389/fped.2023.1147226)

Supplementary Material

Since the sound sources were measured both inside and outside the incubator box, they were divided into two groups for the t-test: Noise sources inside the incubator that were measured with a microphone outside the incubator and noise sources outside the incubator that were measured with a microphone inside the incubator.

	t	p
Sound source outside measured by microphone inside the incubator	-3.283	0.004

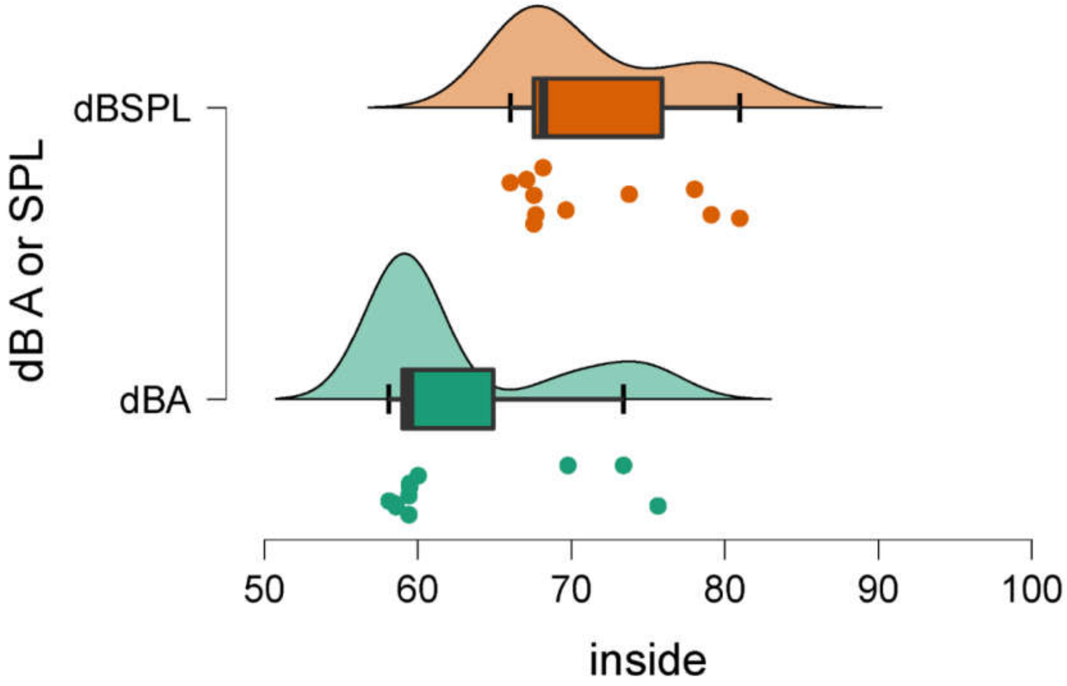

	t	p
Sound source inside measured by microphone outside the incubator	-2.305	0.040

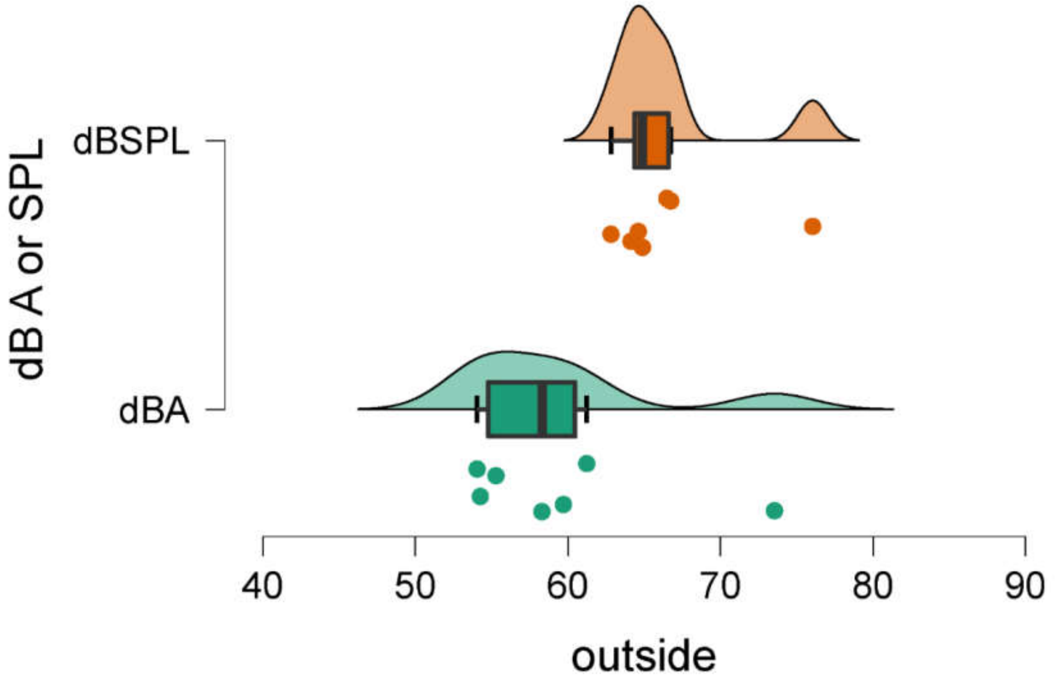

Supplement: Supplementary file 1 [file Datasheet1.pdf]
